# Ultra-sensitive detection of pyridine in water using zinc porphyrin incorporated in a transparent hydrophobic film

**DOI:** 10.1038/s41598-022-09927-x

**Published:** 2022-04-06

**Authors:** Ryo Sasai, Yu-hei Aoyama, Takuya Fujimura

**Affiliations:** Graduate School of Natural Science and Technology, Shimane University, 1060 Nishi-Kawatsu-cho, Matsue, 690-8504 Japan

**Keywords:** Chemistry, Materials chemistry

## Abstract

In this study, we investigated the axial coordination reaction between pyridine and zinc *meso*-tetra(4-sulfonatophenyl)porphyrin (ZnTPPS) incorporated in a transparent layered double hydroxide (LDH) film modified with 1-decanesulfonate (C10S) in an aqueous solution. The apparent equilibrium constant (${K}_{11}$) of the axial coordination reaction between pyridine and ZnTPPS incorporated in the transparent ZnTPPS/C10S/LDH film was approximately 260 times that of the corresponding reaction in an aqueous solution. The hydrophobisation of the LDH interlayer space by C10S, which led to the elimination of water molecules surrounding ZnTPPS and enabled the accumulation of pyridine molecules, was responsible for such a significant increase in the apparent ${K}_{11}$ value. The developed film can detect pyridine in aqueous solutions with ultra-high sensitivity in the order of 10^−5^ mol/L through changes in the colour tone, which is comparable to the molecular detection ability of insect antennae. The sensing response was also observed at pyridine concentrations as low as 10^−9^ mol/L.

## Introduction

Sensor devices that can detect specific chemical compounds with high sensitivity and selectivity are key to achieving the *Sustainable Development Goals* adopted by the United Nations in 2015^1^. Advanced sensors that can detect extremely small amounts of specific chemicals (at nano- and micro-molar concentrations) that are difficult to identify using current sensors, are desirable in various fields, such as non-invasive biological and environmental monitoring. Although sensors based on quartz oscillators and semiconductors that show ultra-high sensitivities towards various chemicals have been developed, the sensors themselves exhibit limited sensitivities and selectivities, and the range of detectable chemicals is not sufficiently diverse^2^. Porphyrin and its derivatives exhibit significantly large extinction coefficients in the visible region, and they exhibit versatile functions, such as light harvesting, oxygen transport, and catalysis in biological systems. Various metal ions can be inserted into the porphyrin ring, and then photophysical/photochemical properties of metalloporphyrin are varied using the inner metal ion of porphyrin. The inner metal ions of metalloporphyrin provide Lewis acid sites, which various basic molecules and ions can coordinate to the centre metal as axial ligands. Subsequently, coordination or exchange of axial ligand results in further changes of the electric structure and photophysical properties of metalloporphyrin. This unique property has motivated several researchers to examine the molecular recognition and sensing by metalloporphyrin. To obtain molecular recognition and sensing system utilizing metalloporphyrins, their synthesis and coordination properties have been reported^3,4^. However, the detection of target chemicals in aqueous media is impeded by the competition between the axial coordination reactions of the target chemicals with the central metal of the metalloporphyrin in water, in addition to the reactions between the target chemicals and water. In such systems, the detection limit can be improved via the hydrophobisation of the metalloporphyrin environment.

In this context, we have previously developed functional materials by hybridizing functional molecules, such as dyes, with ion-exchangeable layered inorganic compounds modified with amphipathic molecules^5–21^. More specifically, an R6G/C16TMA/Lap hybrid material comprising rhodamine 6G (R6G) and laponite clay (Lap) modified by the hexadecyltrimethylammonium (C16TMA) cation, retained the luminous properties of R6G under both dry and wet conditions^7^. Through the use of hexyltrimethylammonium (C6TMA) cations with shorter alkyl chains instead of C16TMA, the luminous properties of R6G in the R6G/C6TMA/Lap hybrid material were varied under dry and wet conditions^8^. These results indicate that the adsorption of water molecules in the interlayer space of Lap modified by alkyltrimethylammonium cations can be controlled by tuning the hydrophobicity of amphipathic molecules. Therefore, the axial coordination reaction of the target chemicals with the central metal of the metalloporphyrin can be expected to proceed in the interlayer space of ion-exchangeable layered inorganic compounds modified with amphipathic molecules bearing long alkyl chains without competition from water molecules.

Herein, we report the preparation of a transparent solid film by hybridizing zinc-*meso*-tetra(4-sulfonatophenyl)porphyrin (ZnTPPS) and 1-decanesulfonate (C10S) with a layered double hydroxide (LDH), which is a layered inorganic compound similar to anion-exchangeable clay. Hereafter, we refer to the prepared transparent solid film as the ZnTPPS/C10S/LDH transparent solid film. Subsequently, we immersed the film in aqueous pyridine solutions of various concentrations and investigated the axial coordination reaction between pyridine and ZnTPPS in the ZnTPPS/C10S/LDH transparent solid film by UV − Vis absorption spectroscopy.

## Results and discussion

**Figure 1:**
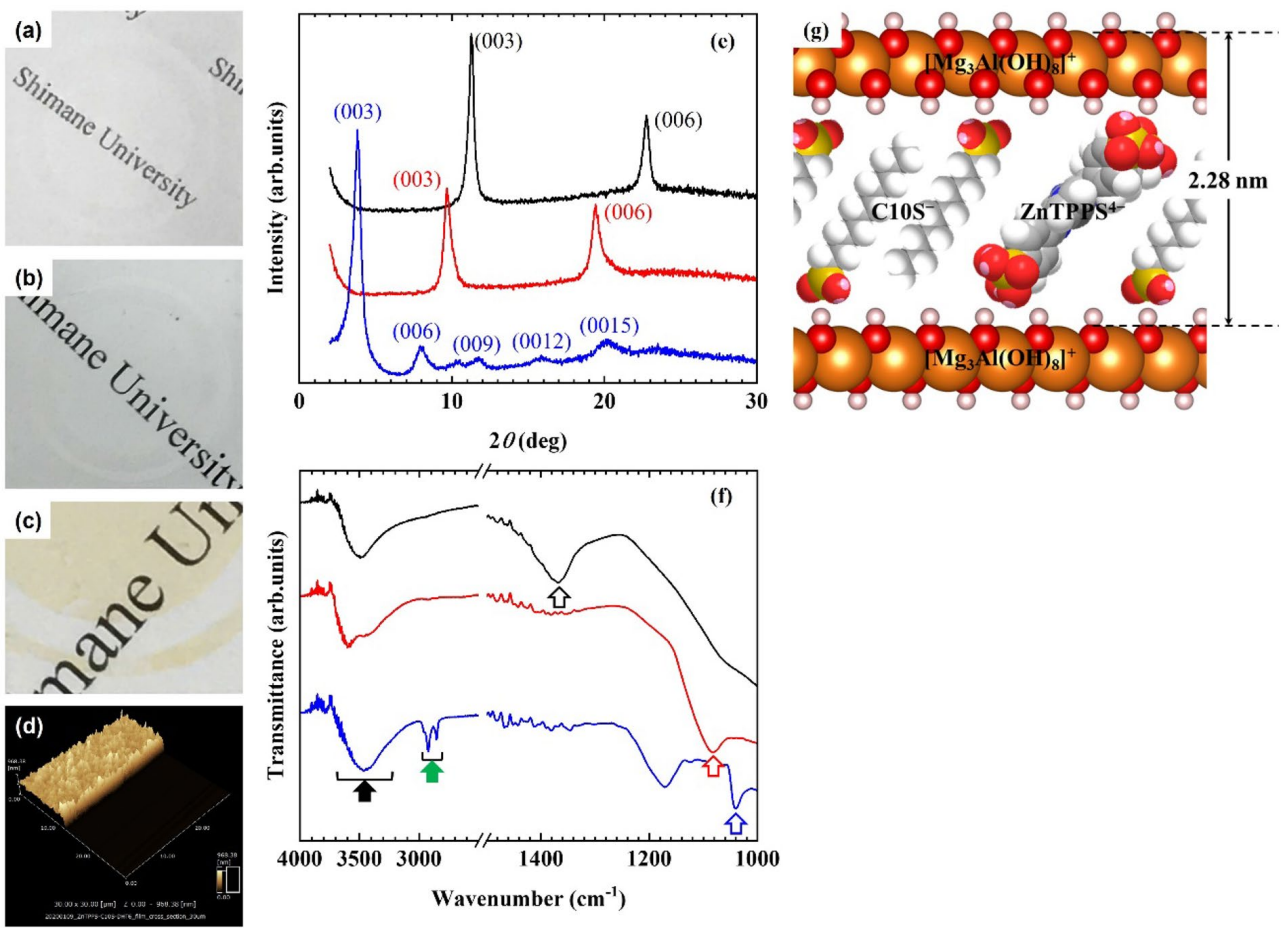
Photographic images of the **(a)** CO_3_^2−^-LDH, **(b)** ClO_4_^−^-LDH, and **(c)** ZnTPPS/C10S/LDH transparent films. **(d)** AFM image of the transparent ZnTPPS/C10S/LDH film. **(e)** XRD patterns of the CO_3_^2−^-LDH (black solid line), ClO_4_^−^-LDH (red solid line), and ZnTPPS/C10S/LDH transparent films (blue solid line). **(f)** FT-IR spectra of the CO_3_^2−^-LDH (black solid line), ClO_4_^−^-LDH (red solid line), and transparent ZnTPPS/C10S/LDH films (blue solid line). Arrows indicate the peaks originating from the hydroxyl (black), carbonate (black (open)), methylene (green), perchlorate (red (open)) and sulfonate (blue (open)) groups. **(g)** Schematic structural model of the ZnTPPS/C10S/LDH materials.

As shown in Fig. 1, all the films prepared using the procedures described in this study exhibited sufficient transparency. The incorporation of ZnTPPS in the transparent ZnTPPS/C10S/LDH film was confirmed by the appearance of a light orange colour. Furthermore, the fraction of ZnTPPS incorporated in the transparent ZnTPPS/C10S/LDH film was estimated to be 3.8% based on an analysis of the anion-exchange capacity of the immersion solution obtained after the immersing operation. According to the results of atomic force microscopy (AFM) studies, the average thickness of the transparent ZnTPPS/C10S/LDH film was 579.3 nm. From the X-ray diffraction (XRD) patterns of the CO_3_^2−^-LDH, ClO_4_^−^-LDH, and transparent ZnTPPS/C10S/LDH films (Fig. 1e), the *d*_003_ values of the CO_3_^2−^-LDH, ClO_4_^−^-LDH, and transparent ZnTPPS/C10S/LDH films were found to be 0.785, 0.954, and 2.28 nm, respectively, indicating that the target anions were incorporated in the interlayer spaces of the LDHs in the transparent films. In addition, the Fourier-transform infrared (FT-IR) spectra of the CO_3_^2−^-LDH, ClO_4_^−^-LDH, and ZnTPPS/C10S/LDH transparent films are shown in Fig. 1f. The absorption band at 1368 cm^−1^ corresponding to the carbonate anions of the transparent CO_3_^2−^-LDH film was absent in that of the spectrum for the transparent ClO_4_^−^-LDH film; instead, an absorption band ascribed to the perchlorate anions was observed. Moreover, in the FT-IR spectrum of the transparent ZnTPPS/C10S/LDH film, absorption bands corresponding to the hydroxyl groups from the LDH layer surface and the hydrated water molecules appeared at ~ 3500 cm^−1^, while bands originating from the methylene groups of C10S appeared at 2923 and 2855 cm^−1^, and those attributed to the sulfonate groups of C10S and ZnTPPS were observed at 1040 cm^−1^. These results indicated the successful synthesis of the transparent ZnTPPS/C10S/LDH film by the procedure reported herein. The schematic structural model of ZnTPPS/C10S/LDH shown in Fig. 1(g) was therefore proposed based on the molecular sizes of ZnTPPS (1.90 nm) and C10S (1.58 nm), the thickness of the LDH monolayer (0.48 nm), and the *d*_003_ value (2.28 nm).

**Figure 2:**
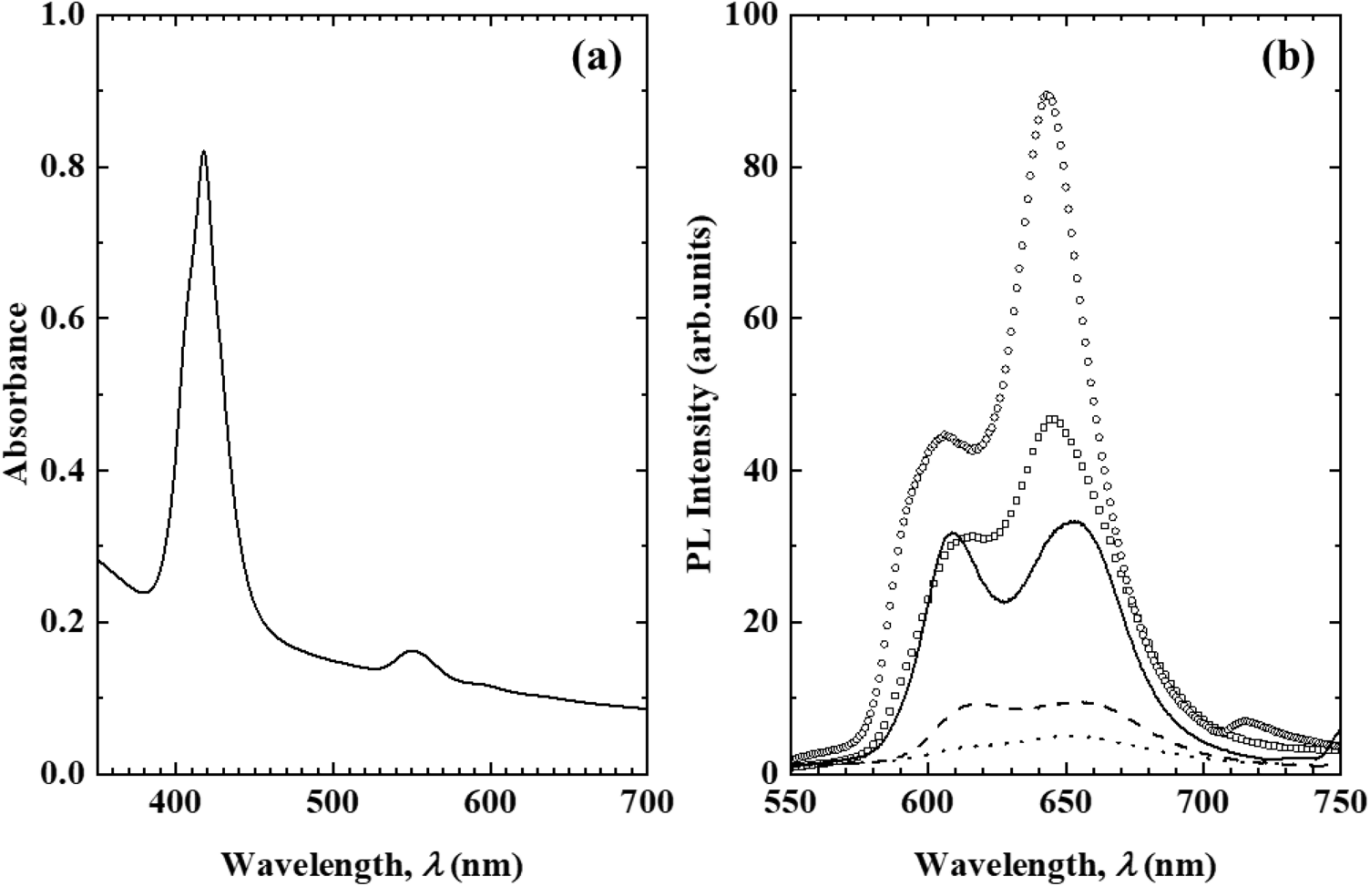
**(a)** Absorption spectrum of the transparent ZnTPPS/C10S/LDH film. **(b)** PL spectra of the transparent ZnTPPS/C10S/LDH film at excitation wavelengths of 410 (open square), 420 (open circle), 430 (solid line), 440 (broken line), and 450 (dotted line) nm.

Figure 2 shows the absorption and photoluminescence (PL) spectra of the transparent ZnTPPS/C10S/LDH film. The Soret and Q bands originating from ZnTPPS in the absorption spectrum of the transparent ZnTPPS/C10S/LDH film (Fig. 2a) indicated that the majority of ZnTPPS molecules incorporated in the interlayer space of the LDH in the transparent film existed as monomers. Furthermore, the PL spectra of the transparent ZnTPPS/C10S/LDH films showed different shapes depending on the excitation wavelength, thereby indicating that a fraction of the ZnTPPS incorporated in the interlayer space of the LDH in the transparent film formed aggregates.

**Figure 3:**
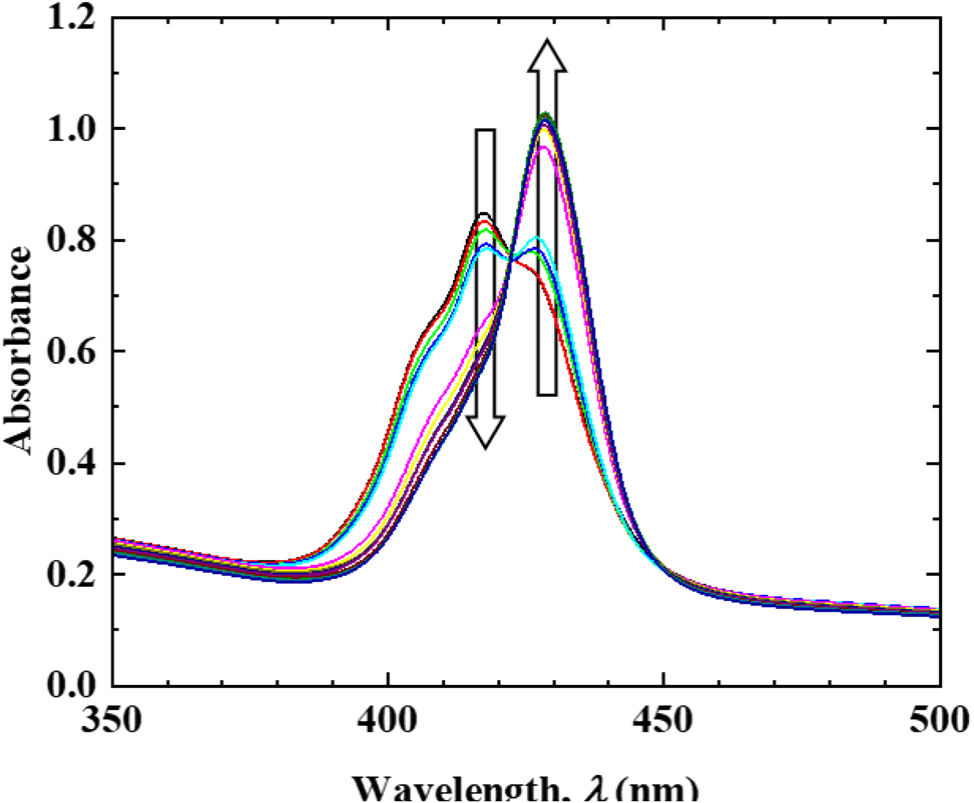
Absorption spectra of the ZnTPPS/C10S/LDH transparent film after immersion in solutions of various [Pyridine] ([Pyridine] = 12.3 µmol/L to 0.123 mol/L).

Figure 3 depicts the absorption spectra of the transparent ZnTPPS/C10S/LDH film immersed in aqueous pyridine solutions of various concentrations ([Pyridine] ranging from 12.3 μmol/L to 0.123 mol/L). As shown, upon increasing [Pyridine], the intensity of the absorption peak at 417 nm gradually diminished with the concomitant appearance of a new peak at 428 nm. Moreover, an isosbestic point was observed at 422 nm. Such spectral changes are characteristic of reactions between two states. A similar change in the Soret band was observed during the formation of a 1:1 complex (Py-ZnTPPS) between ZnTPPS and pyridine (Py) as outlined in Eq. (1). The reaction was performed in an aqueous medium (Fig. S1 in Supporting Information), and upon immersing the transparent ZnTPPS/C10S/LDH film in an aqueous solution of pyridine, the ZnTPPS in the interlayer space of C10S/LDH formed Py-ZnTPPS. As mentioned above, because a fraction of the ZnTPPS was incorporated in the interlayer space of the LDH, the change in absorption spectrum shown in Fig. 3 includes the change due to the dissociation of the aggregate on account of the Py-ZnTPPS formation interaction.

1 $${\text{ZnTPPS }} + {\text{ Py}} \rightleftharpoons {\text{Py}} - {\text{ZnTPPS}}$$

**Figure 4:**
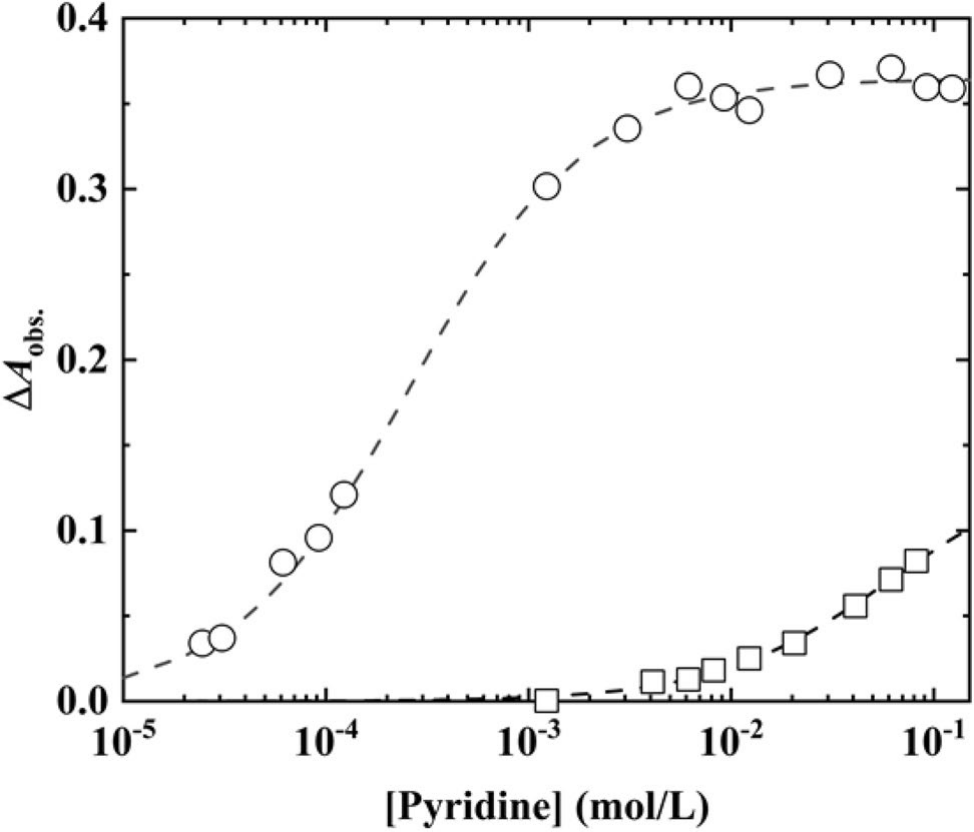
Dependence of Δ*A*_obs._ on [Pyridine] for the transparent ZnTPPS/C10S/LDH film (open circle, 429 nm) and ZnTPPS (open square, 425 nm) in an aqueous solution. The broken lines represent the fitting curves estimated using Eq. (1).

The dependence of $\Delta {A}_{\mathrm{obs}.}$ ($\Delta {A}_{\mathrm{obs}.}={A}_{\mathrm{obs}.}-{A}_{0}$) on [Pyridine] is outlined in Fig. 4, where ${A}_{\mathrm{obs}.}$ and ${A}_{0}$ are the absorbances corresponding to the Soret bands in the presence and absence of pyridine, respectively. The $\Delta {A}_{\mathrm{obs}.}$ value of the transparent ZnTPPS/C10S/LDH film was monitored at [Pyridine] values ranging from 10^−3^ to 10^−5^ mol/L in the aqueous ZnTPPS solution. The $\Delta {A}_{\mathrm{obs}.}$ values indicate that the rate of formation of the Py-ZnTPPS complex was enhanced by incorporating ZnTPPS in the interlayer space of the LDH modified by C10S. Furthermore, the transparent ZnTPPS/C10S/LDH film was capable of detecting pyridine at low concentrations in the order of μmol per litre. In addition, it was found that the pyridine detection sensitivity of the transparent ZnTPPS/C10S/LDH film in the aqueous solution did not reach that of the Re-based molecular trap (i.e., 72 nmol/L) employing an evanescent wave infrared chemical sensing method, which is recognized as a high-sensitivity detection method, and was reported by Huang et al.^22^. However, the pyridine detection sensitivity of the transparent ZnTPPS/C10S/LDH film in the aqueous solution was significantly higher than those of the ZnS nanoparticles reported by Li et al. (6.76 × 10^−5^ mol/L)^23^ and the CuI-coated Cu foil reported by Lv et al. (1.0 × 10^−3^ to 5.0 mol/L)^24^. Therefore, the results presented herein indicate for the first time that extremely sensitive molecular detection can be realised by strategically designing the interlayer space of LDH to facilitate the axial coordination reaction of metallic porphyrins. Therefore, the developed transparent ZnTPPS/C10S/LDH film serves as a highly sensitive film-based device for pyridine detection.

**Figure 5:**
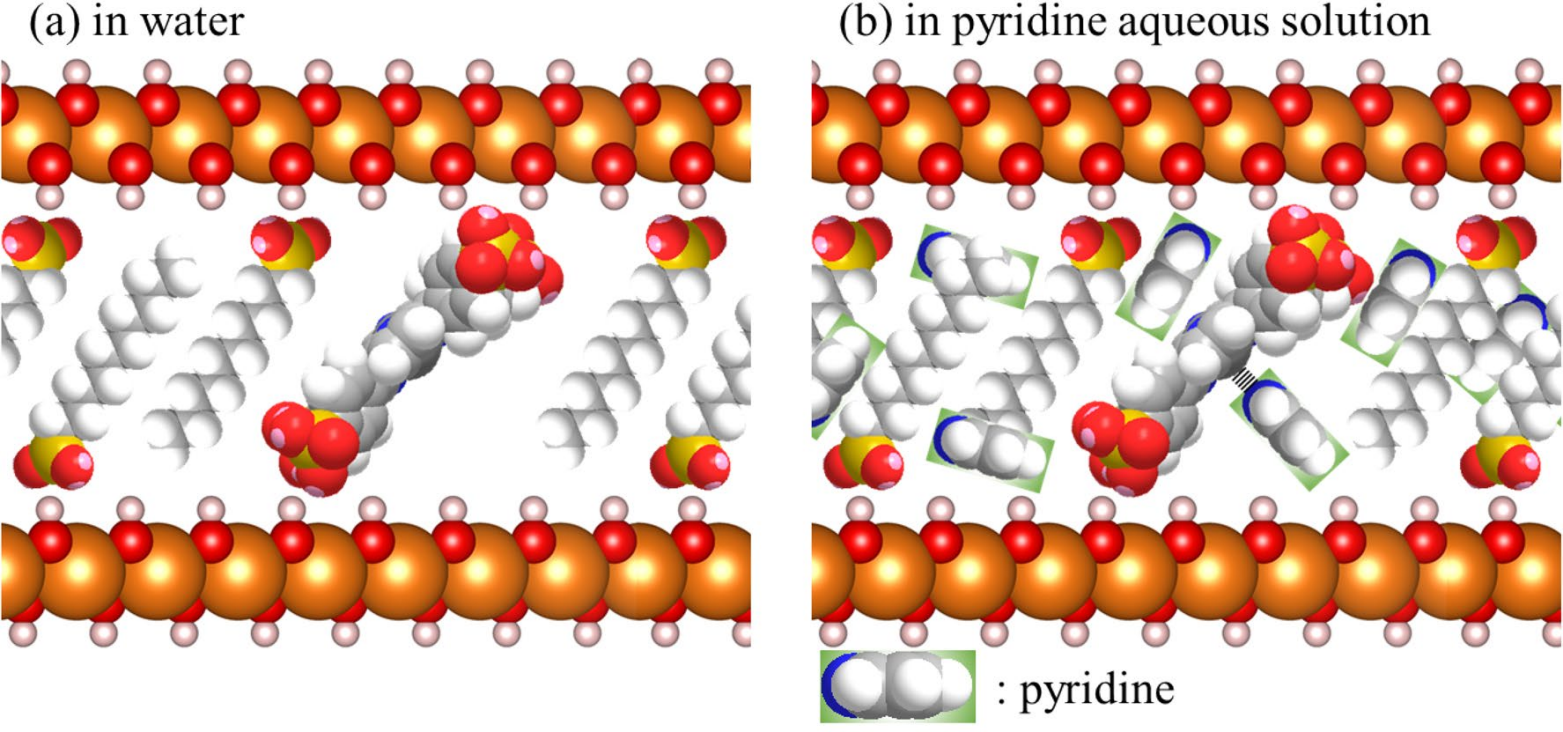
Schematic structural model of the ZnTPPS/C10S/LDH materials in the **(a)** absence and **(b)** presence of pyridine in an aqueous solution.

The dependence of $\Delta {A}_{\mathrm{obs}.}$ on [Pyridine] can therefore be expressed by Eq. (2):

2 $$\Delta {A}_{\mathrm{obs}.}=\frac{b\Delta {\varepsilon }_{11}}{2{K}_{11}}\left[1+{K}_{11}{\left[\mathrm{Py}\right]}_{0}+{K}_{11}{\left[\mathrm{ZnTPPS}\right]}_{0}-{\left\{{\left(1+{K}_{11}{\left[\mathrm{Py}\right]}_{0}+{K}_{11}{\left[\mathrm{ZnTPPS}\right]}_{0}\right)}^{2}-4{K}_{11}^{2}{\left[\mathrm{Py}\right]}_{0}{\left[\mathrm{ZnTPPS}\right]}_{0}\right\}}^\frac{1}{2}]\right],$$

where

3 $$\Delta {\varepsilon }_{11}= {\varepsilon }_{11}-{\varepsilon }_{\mathrm{ZnTPPS},}$$

where ${K}_{11}$ is the ‘apparent’ equilibrium constant of the Py-ZnTPPS formation interaction (reaction (1)) in the interlayer space of ZnTPPS/C10S/LDH. Although Eq. (2) is valid a uniform system, the data obtained in this study was analysed as a pserdu-uniform system under the assumption that the interlayer space of ZnTPPS/C10S/LDH are sufficiently wide for the absorption of pyridine in ZnTPPS/C10S/LDH. The $b$, ${\left[\mathrm{Py}\right]}_{0}$, ${\left[\mathrm{ZnTPPS}\right]}_{0}$, ${\varepsilon }_{11}$, and ${\varepsilon }_{\mathrm{ZnTPPS}}$ are light path length (1 cm), initial [Pyridine], initial concentration of ZnTPPS, and molar extinction coefficients (L∙mol^−1^∙cm^−1^) of Py-ZnTPPS and ZnTPPS, respectively. To analyse the various data related to the transparent ZnTPPS/C10S/LDH film, uniform dissolution of ZnTPPS in the aqueous solution (2 mL) was assumed. The [Pyridine] dependence of $\Delta {A}_{\mathrm{obs}.}$ was analysed by the nonlinear least squares method using Eq. (2). A significantly high apparent ${K}_{11}$ value of 4.17 × 10^3^ L/mol was obtained for the Py-ZnTPPS formation interaction in the transparent ZnTPPS/C10S/LDH film, which was much higher than that of the Py-ZnTPPS formation interaction in the aqueous solution. The formation of Py-ZnTPPS in the aqueous solution competes with the reaction between pyridine and water. Notably, the hydrophobic interlayer space of ZnTPPS/C10S/LDH does not permit water adsorption. In contrast, pyridine is adsorbed and concentrated in the interlayer space of ZnTPPS/C10S/LDH (c.f., Fig. 5). Therefore, ZnTPPS can easily form Py-ZnTPPS in the transparent ZnTPPS/C10S/LDH film in the absence of competition from water molecules. As a result, the ${K}_{11}$ value of the Py-ZnTPPS formation reaction increased significantly. Moreover, it was found that the detection limit of the pyridine concentration was improved by at least two order of magnitude.

**Figure 6:**
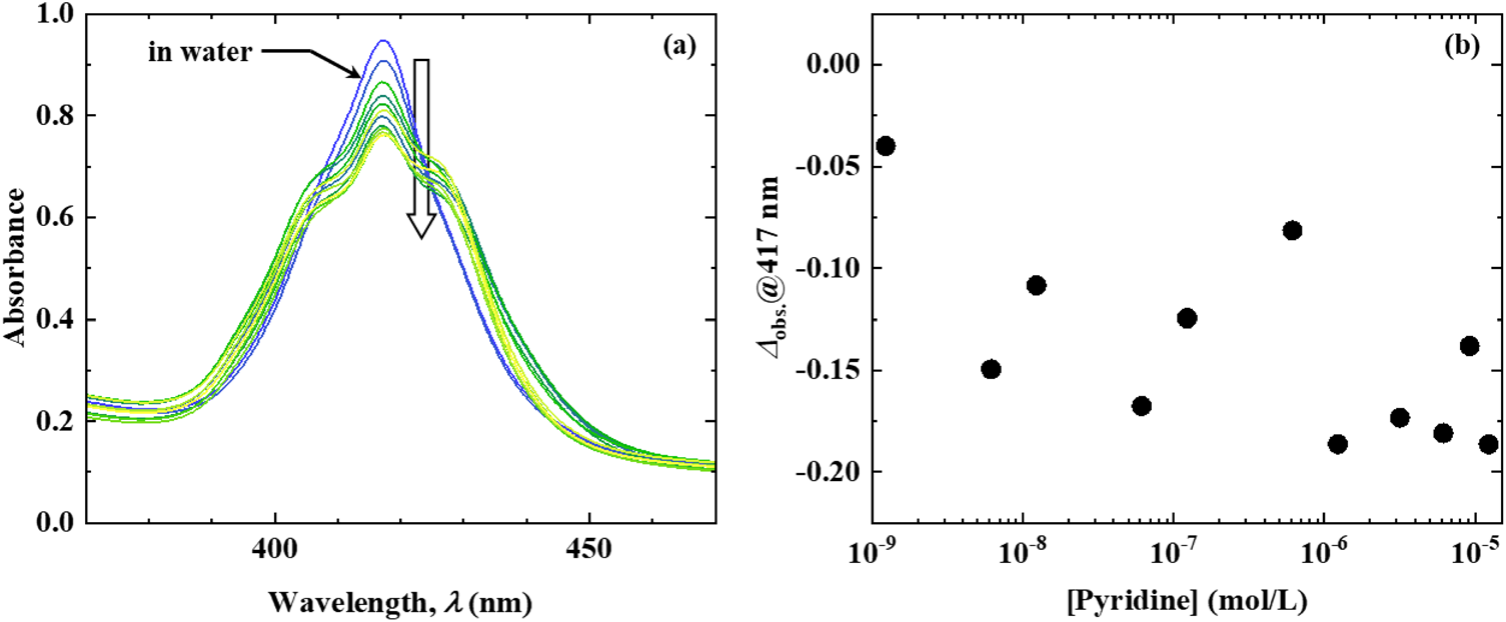
**(a)** Absorption spectrum of the transparent ZnTPPS/C10S/LDH film after immersion in various solutions of low [Pyridine] ([Pyridine] = 1.23 nmol/L to 12.3 µmol/L). **(b)** Dependence of Δ*A*_abs._ on [Pyridine] at 417 nm.

Figure 6a shows the absorption spectra of the transparent ZnTPPS/C10S/LDH film immersed in aqueous pyridine solutions with extremely low [Pyridine] (ranging from 1.23 nmol/L to 12.3 μmol/L). A change in the absorbance at 417 nm was noted at a [Pyridine] as low as 1.23 nmol/L. Moreover, the absorbance at 417 nm decreased with increasing [Pyridine], while the $\Delta {A}_{\mathrm{obs}.}$ value at 417 nm decreased exponentially with increasing [Pyridine] (Fig. 6b). According to these results, the transparent ZnTPPS/C10S/LDH film can quantitatively detect pyridine in aqueous solutions at extremely low [Pyridine] (in the order of nanomoles per litre), indicating that the pyridine sensing performance of the transparent ZnTPPS/C10S/LDH film surpassed the molecular detection abilities of previously reported sensors^22,23^. Furthermore, the pyridine detection ability of this transparent ZnTPPS/C10S/LDH film in an aqueous solution is comparable to that of the evanescent wave infrared chemical sensing method that can achieve highly sensitive detection. Although the isosbestic point was not observed at extremely low [Pyridine], new absorption bands appeared at approximately 405 and 425 nm. This may be attributed to the changes in both the polarity of the interlayer space of ZnTPPS/C10S/LDH and the intermolecular interactions among the ZnTPPS molecules induced by the entry of pyridine into the interlayer space of ZnTPPS/C10S/LDH, and not the formation of Py-ZnTPPS. However, further investigation of band assignment is necessary due to the fact that the mechanistic details underlying this behaviour remain unclear. In future, various experiments will be performed to clarify the pyridine detection mechanism at extremely low [Pyridine] and to improve the quantitative detection of pyridine in aqueous solutions at extremely low [Pyridine].

## Conclusions

In this study, we investigated the pyridine detection performance of a transparent ZnTPPS/C10S/LDH film, which was prepared by hybridizing zinc *meso*-tetra (4-sulfonatophenyl)porphyrin (ZnTPPS) and 1-decanesulfonate (C10S) with a transparent layered double hydroxide (LDH) film prepared by the filtration membrane transfer method in an aqueous solution. The ZnTPPS incorporated in the transparent ZnTPPS/C10S/LDH film formed a 1:1 complex with pyridine, and the formation of Py-ZnTPPS on the transparent ZnTPPS/C10S/LDH film occurred at a pyridine concentration (12.3 μmol/L) that was 100 times lower than that at which the Py-ZnTPPS complex was formed in an aqueous solution. The apparent equilibrium constant $({K}_{11})$ of the Py-ZnTPPS formation reaction on the transparent ZnTPPS/C10S/LDH film in an aqueous solution was 260 times that observed in an aqueous solution of ZnTPPS. Furthermore, changes were observed in the absorption spectra of the transparent ZnTPPS/C10S/LDH film at extremely low pyridine concentrations (i.e., between 1.23 nmol/L and 12.3 μmol/L), which demonstrated the ability of the transparent ZnTPPS/C10S/LDH film to quantitatively detect pyridine at extremely low concentrations. These results therefore indicate that the synthesized transparent ZnTPPS/C10S/LDH film is an effective molecular sensing material for the quantitative detection of pyridine in aqueous solutions with ultra-high sensitivity, as evidenced by the change in the absorbance at 417 nm (Soret band). The propensity of metalloporphyrins to exhibit specific axial coordination patterns depending on the central metal species has thus been exploited to prepare a transparent film by hybridizing a metalloporphyrin with an LDH modified by amphiphilic compounds for the selective axial coordination reaction with pyridine, a toxic species. Such films have the potential to detect toxic coordinate compounds of low polarity in various media. Based on the results presented herein, we are investigating the highly sensitive and selective detection of toxic coordination molecules, such as amines, thiols, and inorganic gases using metalloporphyrins with various central metal atoms, such as Fe, Co, and precious metals incorporated in the interlayer space of LDH. In the future, we aim to develop improved design strategies for the construction of high-sensitivity molecular detection materials using the interlayer 2D nano-space of LDHs.

## Materials and methods

### Materials

The LDH ([Mg_3_Al(OH)_8_](CO_3_)_0.5_∙*n*H_2_O, anion-exchange capacity (AEC): 3.25 mmol/g) was gifted by Kyowa Chemical Industry Co., Ltd. (Sakaide, Japan). ZnTPPS (Frontier Scientific Chemicals, Utah, USA) was used as an anionic metalloporphyrin dye without further purification. C10S (Tokyo Chemical Industry Co., Ltd. Tokyo, Japan) was used as a modifier for the LDH interlayer space without further purification. NaCl (FUJIFILM Wako Pure Chemicals Corporation, Osaka, Japan), CH_3_COOH (AcOH; Nacalai Tesque, Inc. Kyoto, Japan), CH_3_COONa∙3H_2_O (AcONa, Kishida Chemical Co., Ltd. Osaka, Japan), CH_3_CH_2_COONa∙3H_2_O (PrONa, Tokyo Chemical Industry Co., Ltd. Tokyo, Japan), ethanol (EtOH: Fujifilm Wako Pure Chemicals Corporation Osaka, Japan), concentrated sulfuric acid (FUJIFILM Wako Pure Chemicals Corporation Osaka, Japan), methanol (MeOH: Fujifilm Wako Pure Chemicals Corporation, Osaka, Japan), and HClO_4_ (Kanto Chemical Co., Inc. Tokyo, Japan) were used as received without further purification. Pyridine (Fujifilm Wako Pure Chemicals Corporation, Osaka, Japan) was used as the target compound. A cover glass (dimensions: 24 mm × 24 mm, thickness: 0.13–0.17 mm, Matsunami Glass Ind., Ltd. Kishiwada, Japan) was used as the glass substrate. The glass substrate was sonicated in water for 30 min and then treated with concentrated sulfuric acid overnight at 25 ℃. Subsequently, the glass substrate was washed with a sufficient amount of water. The water used in this research was Milli-Q water obtained from a Direct-Q UV system (Merck KGaA, Damstadt, Germany).

### Preparation of transparent LDH films based on C10S and ZnTPPS

A flow chart of the procedure employed to prepare the transparent LDH film based on C10S and ZnTPPS (transparent ZnTPPS/C10S/LDH film) is shown in Fig. S2 in the Supporting Information. An aqueous dispersion of the LDH nanosheet was initially prepared by dispersing the LDH powder-propionic acid mixture (PrO^−^-LDH, 10 mg) in Milli-Q water (20 mL) under vigorous stirring. The PrO^−^-LDH species was synthesized via the NaCl/acetate-buffer decarbonation and anion-exchange methods in the presence of PrONa, as described in the literature^25^. The transparent LDH film bearing carbonate anions (transparent CO_3_^2−^-LDH film) was prepared by applying the filtration-film transfer method to an aqueous dispersion of LDH nanosheets (total volume: 2 mL, LDH particle concentration: 0.15 mg/L) according to our previous report^20^. The transparent LDH film bearing perchlorate anions (transparent ClO_4_^−^-LDH film) was prepared by immersing six pieces of the transparent CO_3_^2−^-LDH film (size: 24 × 24 mm) in an aqueous solution of HClO_4_/MeOH (150 mL, [HClO_4_] = 75.2 μmol/L) for 12 h under a dried N_2_ gas flow. The obtained transparent ClO_4_^−^-LDH film was then rinsed with MeOH and dried under reduced pressure. Subsequently, the transparent ClO_4_^−^-LDH film was immersed in a formamide solution containing both ZnTPPS and C10S (10 mL, [ZnTPPS] = 2.35 mmol/L, [C10S] = 2.35 mmol/L), which was purged with dried N_2_ gas (0.5 L/min) for 3 min, and stirred vigorously for 72 h at 25 ℃. Thereafter, the obtained transparent LDH film based on C10S and ZnTPPS (i.e., the transparent ZnTPPS/C10S/LDH film) was rinsed with EtOH and dried under reduced pressure. After each anion-exchange treatment it was confirmed that the transparent film did not peel off.

### Pyridine detection ability of the transparent ZnTPPS/C10S/LDH film in aqueous media

The pyridine detection ability of the transparent ZnTPPS/C10S/LDH film was evaluated as follows. Initially, the dried transparent ZnTPPS/C10S/LDH film was immersed in aqueous pyridine solutions of various concentrations ([Pyridine] = 0–0.123 mol/L) for 2 min (step 1). After removing the transparent ZnTPPS/C10S/LDH film from the aqueous pyridine solution, the film was dried by blowing with dried N_2_ gas (step 2). The absorption spectrum of the transparent ZnTPPS/C10S/LDH film was then obtained using a UV–Vis spectrophotometer (V-670, JASCO, Tokyo, Japan) (step 3). The transparent ZnTPPS/C10S/LDH film was rinsed twice with EtOH and then dried by blowing with dried N_2_ gas (step 4). Steps 1–4 were repeated at different [Pyridine]. As a reference, the absorption spectra of ZnTPPS in aqueous pyridine solutions with various [Pyridine] were also analysed.

### Characterization

The XRD analysis of each transparent film was performed on a MiniFlex II powder X-ray diffractometer (Rigaku, Tokyo, Japan) equipped with an Ni-filtered Cu-Kα radiation source (30 kV, 15 mA; scan rate: 1°/min; sampling step: 0.02°). Infrared spectroscopy studies were performed on each transparent film using the attenuated total reflection (ATR) method on an FT/IR 6100 spectrometer (JASCO, Tokyo, Japan). The amount of ZnTPPS incorporated in the transparent ZnTPPS/C10S/LDH film was determined from the absorption spectrum of its dimethyl sulfoxide (DMSO) solution, which was prepared by dissolving the residual ZnTPPS collected by evaporation in DMSO. The PL spectra of the transparent ZnTPPS/C10S/LDH film were measured using a spectrofluorometer (FP-6600, JASCO, Tokyo, Japan) at 25 ℃ under ambient conditions. The AFM images were acquired using a scanning probe microscope (SPM-9700, Shimadzu, Kyoto, Japan).

## Supplementary Information

Supplementary Figures.
