# Supplementary material for: Ultra-sensitive detection of pyridine in water using zinc porphyrin incorporated in a transparent hydrophobic film
Source: Sci Rep. 2022 Apr 6;12:5815. doi: 10.1038/s41598-022-09927-x (PMC8987095; doi:10.1038/s41598-022-09927-x)
Supplement: Supplementary file 1 — Supplementary Figures. [file 41598_2022_9927_MOESM1_ESM.docx]

**Supporting Information**


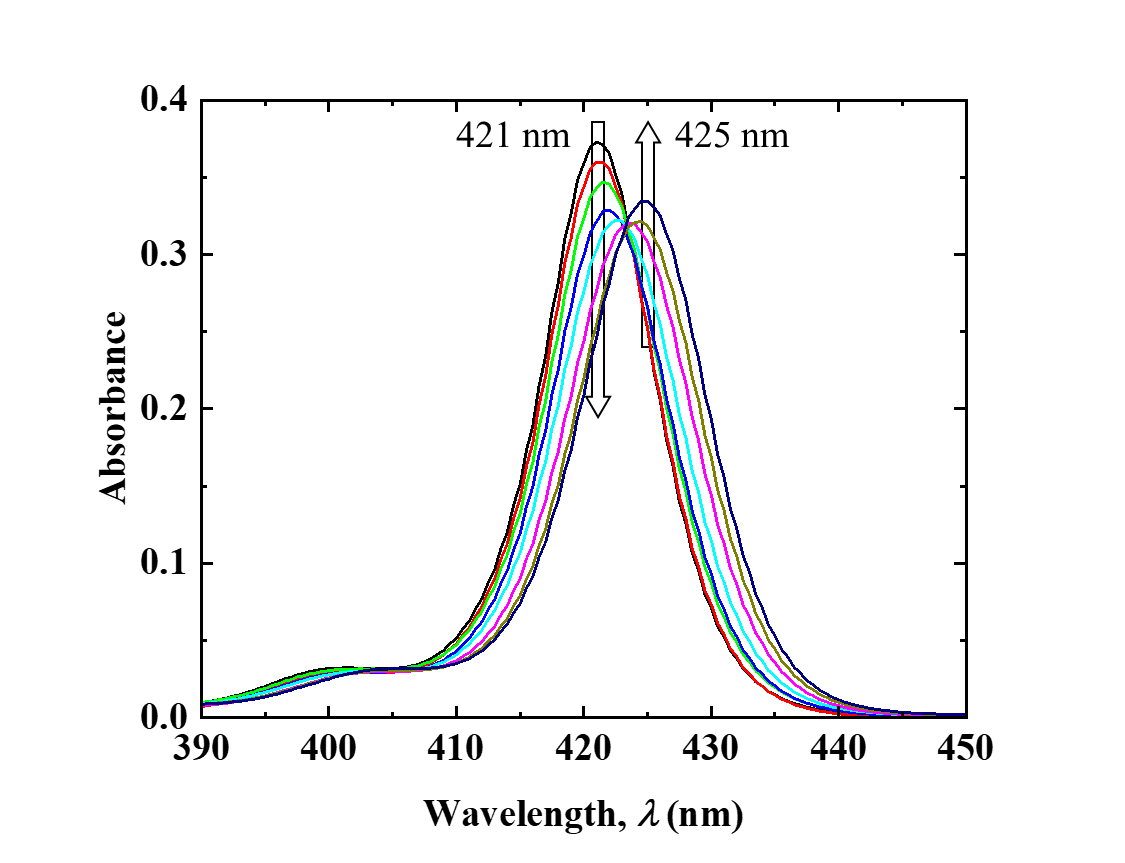


**Figure S1.** Absorption spectra of the ZnTPPS in aqueous solutions of various [Pyridine] (0 – 8.20 × 10^−2^ mol/L).


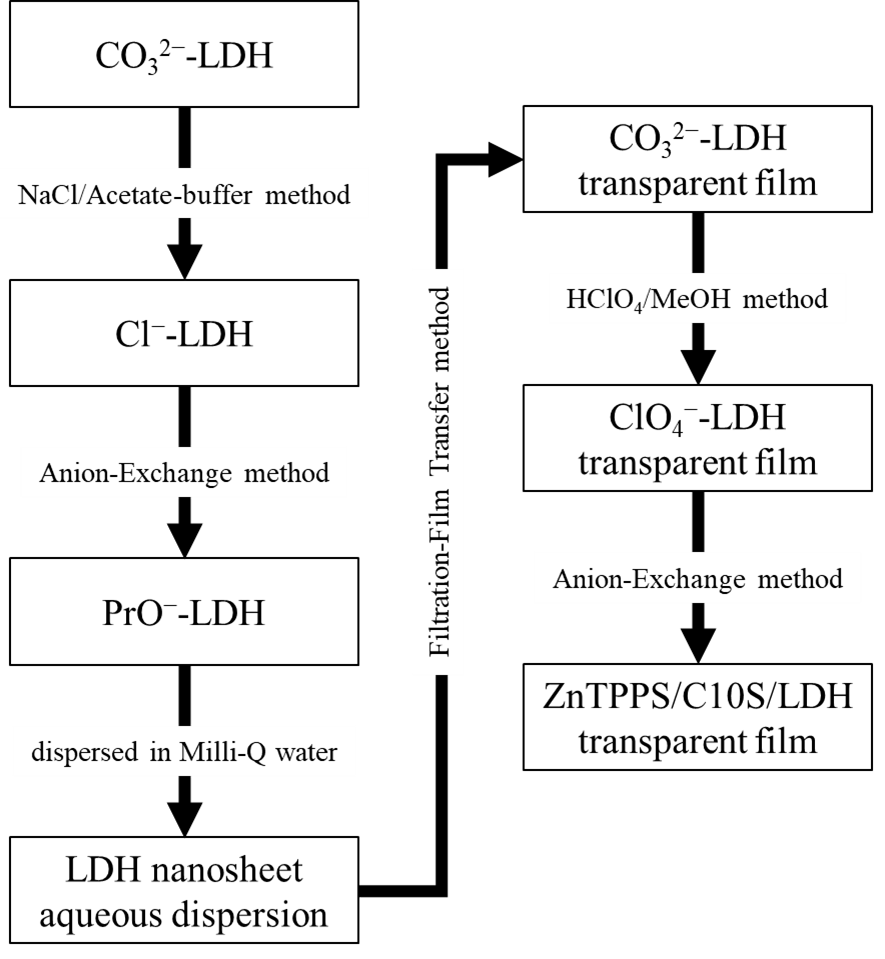
**Figure S2.** Flow chart representing the procedure employed to prepare the transparent ZnTPPS/C10S/LDH film
